# Road Traffic Accidents Presenting to the Emergency Department of a Tertiary Care Center: A Descriptive Cross-sectional Study

**DOI:** 10.31729/jnma.6660

**Published:** 2021-11-30

**Authors:** Rohan Jha, Priya Pathak, Pallavi Koirala, Bishwash Maharjan, Srijana Panthi

**Affiliations:** 1 Department of General Practice and Emergency Medicine, College of Medical Sciences, Bharatpur, Nepal; 2 Kathmandu Institute of Child Health, Kathmandu, Nepal; 3 Department of Community Medicine, College of Medical Sciences, Bharatpur, Nepal

**Keywords:** *injuries*, *Nepal*, *pattern*, *road traffic accidents*

## Abstract

**Introduction:**

Road Traffic Accidents have emerged as the leading cause of mortality and morbidity globally. The burden of road traffic accidents has escalated gradually in Nepal and is a common cause of injury and trauma. The study aims to identify the prevalence of road traffic accidents in the emergency department.

**Methods:**

This descriptive cross-sectional study was conducted among hospital records of cases admitted to the emergency department of tertiary care hospital between March to August, 2020. Ethical approval was taken from the ethical review board of College of Medical Sciences (reference number: 2020-035). Information was collected through pro-forma and hospital records. Convenience sampling was done. The data were entered in the Statistical Package for the Social Sciences version 24 and analysed using descriptive statistics. Point estimate at 95% Confidence Interval was calculated along with frequency and proportion for binary data.

**Results:**

Among 4050 cases presenting to the emergency department, 228 (5.6%) (4.9-6.3 at 95% Confidence Interval) cases of road traffic accidents were seen. The most common injuries involved were soft tissue injury 90 (39.47%) and head injury 77 (33.77%). Most patients admitted to the hospital were male 178 (78.07%) aged 21 to 30 years 79 (41.38%). The vehicles mostly involved in the accidents were motorized two-wheelers 120 (50.6%) and pedestrians 51 (22.4%).

**Conclusions:**

The prevalence of road traffic accidents was similar to the findings from similar studies. Strengthening the capacities of the pre-hospital care and emergency department is necessary along with preventive intervention in public to reduce such health burden.

## INTRODUCTION

Road traffic accidents have emerged as the leading cause of mortality and morbidity globally.^1^. Road injuries and deaths due to road traffic accidents are a major public health problem in developing countries where more than 85% of all deaths and 90% of disability-adjusted — life years were lost from road traffic injuries.^2^

Globally 8.94 deaths per 100,000 population were due to road traffic injuries.^3^ Nepal police have recorded 10030 road traffic accidents in Nepal in the fiscal year of 2076/2077 which is a 17% rise from the previous year.^4,5^ Most road traffic accidents that occur in Nepal are found on highways outside Kathmandu valley^6^. The main risk factor influencing the post-crash outcome of injuries is a delay in the management of injured at the health facility.^7^ Emergency departments is portals for emergency admission to hospitals which provide immediate urgent medical intervention.^8^

This study aims to find the prevalence of road traffic accidents among cases presenting to the department of emergency medicine of the College of Medical Sciences (COMSTH).

## METHODS

This descriptive cross-sectional study was conducted at the department of emergency of COMSTH from March 2020 till August 2020. Ethical approval was taken from the ethical review board of College of Medical Sciences (reference number: 2020-035). Patients presenting to the department of emergency during the study period were included in the study. The study excluded severe trauma cases that are devoid of caregivers and who are unable to give consent. Convenience sampling was done and the the sample size was calculated using the formula,

n = Z^2^ × p × q / e^2^

  = 1.96^2^ × 0.5 × (1-0.5) / 0.02^2^

  = 2401

where,

n= required sample size,
Z= 1.96 at 95% Confidence Interval (CI),
p= past prevalence taken as 50% for maximum sample size,
q= 1-p
e= margin of error, 2%

Adding a 10% non response rate the calculated sample size was 2425. However, 4050 cases were taken.

Information was collected through a pre-tested questionnaire. The observed sample size comprised all the patients presenting to the COMSTH emergency department who were recorded in the emergency records in the study period. Information was collected through a proforma based on the Emergency ticket which was filled according to the duty doctor's observation. Data was collected including patient demographics, accident details, patient symptoms, accident details, patient symptoms, clinical signs, and the outcome. A systematic method of clinical examination of trauma causes in the emergency ticket was used as the basis of data collection for the proformas.

The data collected in the datasheet was recorded and analyzed using the Statistical Package for the Social Sciences version 24. Descriptive statistics were generated for the pattern of admissions, presentation of the type of injury, and the referrals. Point estimate at 95% CI was calculated along with frequency and percentage for binary data.

## RESULTS

A total of 228 (5.629%) (4.9-6.3 at 95% CI) RTA cases were seen among the 4050 cases presenting to the emergency department. Among the RTA victims, 178 (78%) were males and the mean age of the victims was 29.6 years (±15.57). About 95 (41%) of the victims were aged 21 to 30 years followed by the 11-20 years 41 (17.98%) and 31-40 years 39 (17.10%) (Table 1).

**Table 1:** Age and sex distribution of the RTA Victims (n = 228)

Age Groups	Male n (%)	Female n (%)	Total n (%)
0-10	8 (4.49)	9 (18)	17 (7.45)
11-20	28 (15.73)	13 (26)	41 (17.98)
21-30	79 (44.38)	16 (32)	95 (41.66)
31-40	34 (19.1)	5 (10)	39 (17.1)
41-50	10 (5.61)	3 (6)	13 (5.7)
51-60	6 (3.37)	2 (4)	8 (3.5)
>60	13 (7.3)	2 (4)	15 (6.57)
Total	178 (78.07)	50 (21.92)	228 (100)

**Figure 1:**
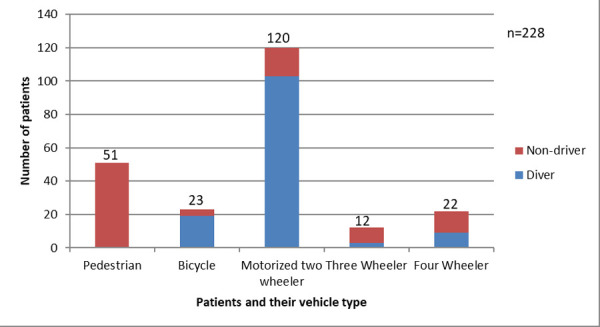
RTA victims and their vehicles.

Motorized two-wheeler (Motorbike and scooter) was the commonest vehicle involved 120 (52.6%) followed by Pedestrian 51 (22.4%) in the accident and bicycle 23 (10.1%) (Figure 1).

Almost 157 (69%) of the cases occurred in monsoon, in July and August and Sunday 51 (22.3%) maximum cases. The majority of the cases occurred during the evening time from 4 PM to 8 PM (Table 2). The average time taken to arrive at the tertiary care hospital after the accident was found to be 94 minutes (± 92) excluding 5% of outliers. The median time taken to reach the hospital is 75 minutes.

**Table 2:** Distribution of Accident time in 24 hour time.

Accident time	n (%)
12 AM-4 AM	11 (4.82)
4AM-8AM	8 (3.5)
8AM-12PM	40 (17.54)
12 PM-4 PM	59 (25.87)
4 PM-8 PM	80 (35.08)
8PM-12AM	30 (13.15)

The RTA victims were assessed by trained medical practitioners on the type of injury sustained. Most of the victims suffered from soft tissue injury 90 (39.47%) followed by head injury 77 (33.77%) and fracture of limb bone 33 (14.47%). Most of the patients after the emergency care in the emergency department were discharged for home care 103 (45.17%) while 26 (11.4%)were admitted in ICU, 77 (33.77%) admitted in a general ward and 8 (3.5%) were referred to an immediate surgical operation (Table 3).

**Table 3:** Distribution of the cases according to their incidence of regional injuries, ICU admission and referral, and Glasgow coma score at the time of presentation.

Characteristics		n (%)
Type of injury sustained by the patient's body part Involved (n=228)	Brought Dead	3 (1.31)
	Head Injury	77 (33.77)
	Spine injury	4(1.75)
	Chest Trauma	13(5.7)
	Fracture of limb bone	33(14.47)
	Pelvic injury	5(2.19)
	Soft tissue injury	90 (39.47)
	Other	3 (1.30)
Patient referral (n=219)	ICU	26 (11.4)
	General ward	77 (33.77)
	Referred to another center	1 (0.43)
	Operational theatre	8 (3.5)
	The patient died in the ER	1 (0.43)
	The patient was brought dead	3 (1.31)
	Discharged	103 (45.17)
Glasgow Coma Scale (n=228)	3-8	14 (6.1)
	9-12	9 (3.9)
	13-15	205 (89.9)
FAST Scan (n=222)	Positive	5 (2.19)
	Negative	217 (95.17)

All the patients with spine and blunt trauma abdomen were admitted to the hospital for further care while 72 (82.8%) of the patients with soft tissue injury, 20 (27.8%) patients with head injury were discharged from the hospital after emergency care (Table 4).

**Table 4:** Distribution of patients by Part involved in injury and admission category (n=219).

Parts involved	Admitted number			Admitted total n (%)	Discharged n (%)	Referred to other centers n (%)
	ICU n (%)	General ward n (%)	Operational theatre n (%)			
Head Injury	17 (23.6)	28 (38.9)	6 (8.3)	51 (70.8)	20 (27.8)	1 (1.4)
Spine injury	1 (33.3)	2 (66.7)	0 (0)	3 (100)	0 (0)	0 (0)
Chest Trauma	2 (15.4)	8(61.5)	0 (0)	10 (76.9)	3 (23.1)	0 (0)
Fracture of limb bone	3 (9.1)	22 (66.7)	1 (3)	26 (78.8)	7 (21.2)	0 (0)
Pelvic injury	2 (50)	1 (25)	0 (0)	3 (75)	1 (25)	0 (0)
Blunt trauma abdomen	0 (0)	0 (0)	1 (100)	1 (100)	0 (0)	0 (0)
Soft tissue injury	1 (1.1)	14 (16.1)	0 (0)	15 (17.2)	72 (82.8)	0 (0)

## DISCUSSION

The study findings showed the most common injuries are soft tissue injury and head injury, most of which didn't require surgical management. The finding aligns with the study conducted by Huang et al. in Kathmandu where the most common injury was also soft injury.^11^ In the study, the victims suffering from a fracture of limb bone accounted for 14.47% which is less than those found by a similar study in Tanzania,^13^ and India^12^ which showed the commonest injury was limb fracture.

This study shows male aged 11 to 40 years have a higher risk of road traffic injuries in Nepal. Other studies were done in western Nepal and India also showed similar results.^9,10^ A study conducted in Kathmandu Valley by Huang L, et al.(2016) concluded that 75% of victims were between 15-49 years old.^11^ A study conducted by Pathak SM. (2012) et al. in a tertiary care hospital in India, concluded that the commonest age group involved in accidents was 20-30 year.^12^ A study conducted in Tanzania in 2014 concluded that the majority of the patients were male (76.6%) and the majority (70.2%) were between 18-45 years of age group.^13^ A study done in Iran about the epidemiological patterns of road traffic crashes from 1996 to 2014 concluded that most of the victims were male aged between 30-39yrs.^14^

According to the Department of Transport, almost 80% of the vehicles registered in Nepal are motorized two-wheelers (motorcycle/ scooter/moped). Motorized two-wheelers comprise the biggest proportion of injuries due to road crashes and the incidences of injuries increases with the use of alcohol and other illicit drugs. Similar findings were found in Iran with 53.4% of road crashes involving motorcycles ^13^ as well as the study was done in 4 low-income countries where motorcycles accounted for 44.7 % of road traffic injuries.^15^ A study conducted by Huang L, et al. in Kathmandu showed two-wheelers vehicles were most frequently involved (67.2%) and pedestrians were the most vulnerable group (33%) which is also supported by this study.

Most of the road traffic accidents occurred during monsoon season when the road is slippery and between 4 PM-8 PM accounting for almost 35% of accidents. Other studies have also found similar findings with excess accidents in monsoon and during dark time occurring between 4 PM-10 PM.^12,14,16^ The burden of road traffic injuries due to RTAs places a huge financial burden on low-income countries like Nepal evidently affecting the productive population and lower socioeconomic group. Nepal has the highest percentage of GDP loss (6.3%) due to road traffic accidents.^12^

Our study could not include the primary causes of road traffic accidents like road condition, vehicle condition, non-use of helmets, and seat belts. Other factors contributing to the total number of fatalities, like the ambulance response times, and the first aid management were not included in the study.

## CONCLUSIONS

The prevalence of road traffic accidents were similar to the findings from similar studies. This study showed that males, young age, consumption of alcohol, drivers, and pedestrians had a higher mortality and morbidity. Most of the deaths occur before the arrival at the hospital. Hence, awareness on good pre-hospital care or first aid care can be effective to reduce the death toll due to RTAs. Head injuries remain the most common and head, spine, and blunt abdomen injuries were the most serious type of trauma encountered at the emergency department, Therefore, the availability of a well-equipped ambulance, trained paramedic human resources, comprehensive trauma team comprising neurosurgical, orthopedics, maxillofacial care professional is essential. Every tertiary care hospital should establish an integrated approach to the management of road traffic injuries.
